# Inflammatory phenotypes in acute respiratory distress syndrome: external validation and model simplification in multicenter cohorts

**DOI:** 10.3389/fmed.2026.1877882

**Published:** 2026-06-24

**Authors:** Yuanyuan Li, Wenqiang Li, Feng Qu, Ping Wei

**Affiliations:** 1 Department of ICU, Jining No.1 People’s Hospital, Jining, Shandong, China; 2 School of Public Health, Shandong Second Medical University, Weifang, Shandong, China

**Keywords:** acute respiratory distress syndrome, external validation, inflammatory phenotype, prognosis, risk stratification

## Abstract

**Background:**

Inflammatory phenotyping represents a promising approach for risk stratification in acute respiratory distress syndrome (ARDS). A previously developed 18-variable model classifies patients into hyperinflammatory and hypoinflammatory phenotypes; however, its generalizability and feasibility for routine implementation remain uncertain. We aimed to externally validate this model and determine whether a simplified 17-variable version, excluding minute ventilation, maintains equivalent prognostic performance.

**Methods:**

This retrospective cohort study was conducted using the Medical Information Market for Intensive Care IV (MIMIC-IV) and electronic intensive care unit (eICU) Collaborative Research Database. In the MIMIC-IV cohort, inflammatory phenotypes were assigned using the original 18-variable model, and their association with 30-day mortality was evaluated using logistic regression. A simplified 17-variable model excluding minute ventilation was also assessed. Model discrimination was compared using the area under the receiver operating characteristic curve (AUC) and DeLong’s test. External validation was performed in the eICU cohort. Sensitivity analyses included multiple imputation in MIMIC-IV and complete-case analysis in the eICU cohort.

**Results:**

In the MIMIC-IV cohort (*n* = 938), the hyperinflammatory phenotype was associated with higher 30-day mortality. The simplified 17-variable model demonstrated near-perfect agreement with the original model (99.9%) and comparable discriminative performance. In the eICU cohort, the hyperinflammatory phenotype remained significantly associated with increased mortality, with moderate discriminative ability. Sensitivity analyses yielded consistent results.

**Conclusion:**

The inflammatory phenotype model demonstrated robust prognostic value across independent ARDS cohorts. A simplified 17-variable model, excluding minute ventilation, maintained an equivalent performance, supporting its potential as a practical and generalizable tool for clinical implementation.

## Introduction

Acute respiratory distress syndrome (ARDS) is a common and life-threatening condition in critically ill patients and associated with substantial morbidity and mortality (1). According to the Berlin definition, ARDS is characterized by acute hypoxemia and bilateral pulmonary infiltrates that cannot be fully explained by cardiac failure or fluid overload (2). Recent updates to the global definition of ARDS have expanded the diagnostic criteria to improve clinical applicability, including the incorporation of high-flow nasal oxygen and alternative oxygenation indices (3, 4). Despite advances in supportive care, overall outcomes have improved only modestly over recent decades. One major challenge is the marked biological and clinical heterogeneity of ARDS, which may obscure treatment effects and contribute to the failure of multiple clinical trials (5).

Accumulating evidence suggests that ARDS can be classified into distinct inflammatory sub-phenotypes, most commonly hyperinflammatory and hypoinflammatory (6). These sub-phenotypes differ in inflammatory profiles, degree of organ dysfunction, and clinical outcomes, with the hyperinflammatory phenotype consistently associated with higher mortality (7, 8). Additionally, differential responses to therapeutic strategies have been observed, highlighting their potential relevance for precision medicine (9).

However, early identification of ARDS subphenotypes has largely relied on circulating biomarkers, such as interleukin-6 and tumor necrosis factor receptors. These biomarkers are not routinely available in clinical practice, thereby limiting real-time bedside applicability (10). To address this limitation, clinical classifiers based on routinely available variables have been developed to enable phenotypic identification without the need for specialized assays (11).

Among these approaches, an artificial intelligence (AI)–based clinical classifier using routinely collected variables has demonstrated promising performance in identifying inflammatory ARDS phenotypes across multiple cohorts (12). Nevertheless, two important issues remain unresolved before widespread clinical implementation. First, external validation in independent real-world populations remains limited. Second, whether a simplified version of the model can retain its performance while improving its feasibility across different clinical settings remains unclear. Although the original classifier demonstrated good performance, certain required variables may not be routinely available across different electronic health record systems. In particular, minute ventilation is often missing or inconsistently recorded in large clinical databases, which may limit the applicability of the original model. Furthermore, minute ventilation is influenced to some extent by local ventilation management strategies and clinician preferences, rather than solely by patient characteristics, which may reduce the model’s transferability across different institutions. Therefore, it remains unclear whether simplifications aimed at practical application can enhance real-world usability while maintaining performance in phenotypic classification and prognostic prediction. Accordingly, we aim to conduct external validation of this classifier and assess whether a simplified model—excluding minute ventilation—can facilitate broader clinical deployment without sacrificing performance.

Therefore, this study aimed to validate an inflammatory phenotype classifier using a large critical care database and evaluate the performance of a simplified model with fewer variables. External validation was additionally performed in an independent multicenter cohort to determine whether model simplification preserved predictive performance while improving clinical applicability.

## Methods

### Study design and data sources

This retrospective multicenter observational study was conducted using the Medical Information Mart for Intensive Care IV (MIMIC-IV, version 3.0) and the electronic intensive care unit Collaborative Research Database (eICU-CRD, version 2.0).

The MIMIC-IV database contains detailed, high-resolution clinical data from patients admitted to the Beth Israel Deaconess Medical Center. Meanwhile, the eICU-CRD is a multicenter database comprising ICU admissions from hospitals across the United States. Both databases are widely used in critical care research and provide complementary settings for model development and external validation.

Access to both databases was obtained via the PhysioNet platform following the completion of the required training.

### Study population

Adult patients aged 18 years admitted to the intensive care unit were screened in both databases. Only patients who were admitted to the ICU for the first time were analyzed, whereas those with an ICU length of stay of <24 h were excluded.

In the MIMIC-IV cohort, patients with ARDS were identified using a stepwise approach consistent with the Berlin Definition. First, patients receiving invasive mechanical ventilation were selected. Among these, eligibility was defined by a PaO_2_/FiO_2_ ratio of ≤300 mmHg under a positive end-expiratory pressure of ≥5 cmH_2_O. The presence of bilateral pulmonary infiltrates on chest imaging within ±48 h and at least one recognized ARDS risk factor was required. Patients with cardiogenic pulmonary edema, acute heart failure, or extracorporeal membrane oxygenation were excluded. After applying these criteria, 940 patients met the definition of ARDS. Patients with missing key variables required for phenotypic classification were excluded, resulting in a final MIMIC-IV cohort of 938 patients.

In the eICU cohort, a simplified operational definition was applied due to the inconsistent availability of detailed imaging data. Adult patients receiving invasive mechanical ventilation were included if they had a PaO_2_/FiO_2_ ratio ≤300 mmHg with a positive end-expiratory pressure of ≥5 cmH_2_O. Patients were also required to have at least one risk factor for ARDS; those with evidence of cardiogenic pulmonary edema were excluded. After exclusion of patients with duplicate records and those with missing key variables required for phenotype classification, the final eICU external validation cohort comprised 1,527 patients.

**Figure 1:**
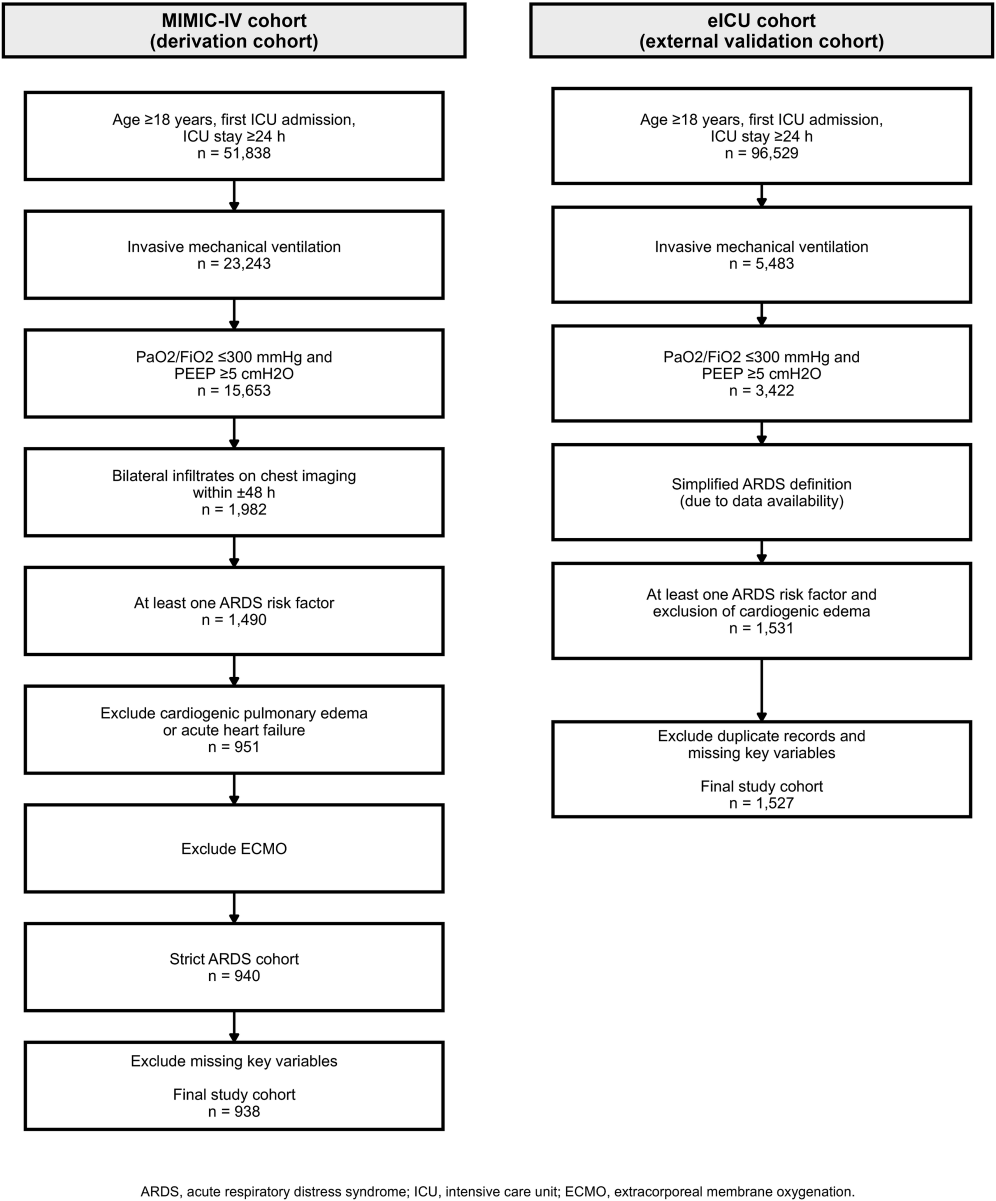
The flowchart illustrates patient screening, inclusion and exclusion criteria, and final cohort selection in the MIMIC-IV and eICU databases. ARDS, acute respiratory distress syndrome; ICU, intensive care unit; ECMO, extracorporeal membrane oxygenation.

ARDS onset was defined as the first time point at which a PaO_2_/FiO_2_ of ≤300 mmHg and a positive end-expiratory pressure of ≥5 cmH_2_O were simultaneously met during invasive mechanical ventilation. The detailed selection process for both cohorts is illustrated in Figure 1.

### Variable extraction and preprocessing

Clinical variables were extracted in accordance with a previously published AI–based clinical classifier for ARDS inflammatory phenotyping (11). This classifier was originally developed using individual patient data from multiple multicenter randomized controlled trials and implemented using an extreme gradient boosting (XGBoost) algorithm.

Eighteen routinely available variables, including laboratory measurements, vital signs, and clinical support parameters, were collected from the original model. To improve the clinical applicability, a simplified 17-variable model was also evaluated by excluding minute ventilation, which is not consistently available in routine clinical practice.

Variable extraction was based on a predefined time window centered on the onset of ARDS. If multiple measurements were available within the extraction window, representative values were selected according to predefined rules, taking into account clinical relevance and consistency with the original classifier framework. Laboratory test results, vital signs, and clinical support variables were all extracted from electronic health record data routinely collected before and after the onset of ARDS. To facilitate reproducibility of the results, the specific extraction windows, source data tables, aggregation rules, unit standardization procedures, and preprocessing methods for each variable are detailed in Supplementary Table S8.

Vasopressor use was treated as a baseline clinical support variable and was defined according to the predefined extraction window surrounding ARDS onset, consistent with the original classifier framework.

All variables were expressed using the same units as the original model to ensure consistency in implementation. These units corresponded to standard SI units or commonly used clinical formats. Unit consistency between the MIMIC-IV and eICU cohorts was carefully verified, and necessary conversions were performed prior to analysis.

The AI-based clinical classifier generated the probability of the hyperinflammatory phenotype. Consistent with the original study (11), a threshold of 0.5 was applied to classify patients into hyperinflammatory (probability ≥ 0.5) and hypoinflammatory (probability < 0.5) phenotypes.

For primary phenotype classification, missing data were not imputed prior to model application. Detailed summaries of variable-specific missingness in the MIMIC-IV and eICU cohorts are provided in Supplementary Tables S6, S10, respectively. Patients with missing key variables required for phenotype assignment were excluded from the analysis. To evaluate the robustness of the findings with respect to missing data, multiple imputation using chained equations was performed as a sensitivity analysis in the MIMIC-IV cohort. By contrast, a complete-case analysis was conducted in the eICU cohort, given the differences in data structure and missingness patterns between the databases.

No multiple imputation was performed in the eICU cohort because the extent and patterns of missing data differed significantly from those observed in MIMIC-IV, and the missing values for multiple variables were associated with data availability and exhibited institutional variability. Therefore, a conservative approach using a complete-cases analysis was adopted for external validation.

Minute ventilation was selected for exclusion because ventilatory variables are more difficult to capture consistently from routine electronic health record (EHR) systems than laboratory and vital sign variables and may be influenced by local ventilation management practices. Previous validation studies of ARDS phenotype classifier models have highlighted that ventilatory variables are harder to extract reliably from EHR data, potentially limiting model portability and implementation across institutions. Therefore, minute ventilation was considered a potential barrier to the broader clinical application of the original classifier and was removed in the simplified model (12). A complete variable extraction protocol for both the original 18-variable classifier and the simplified 17-variable classifier is provided in Supplementary Table S8.

### Inflammatory phenotype classification

Inflammatory phenotypes in the MIMIC-IV cohort were initially assigned using a previously published 18-variable AI-based clinical classifier (11). The original model was applied as a fixed, pretrained model without retraining or recalibration.

To enhance clinical applicability, a simplified 17-variable model was developed by excluding minute ventilation. During model development, phenotype assignments generated by the original 18-variable model in the MIMIC-IV cohort were used as reference labels. The 17-variable model was subsequently trained in the MIMIC-IV cohort using an extreme gradient boosting (XGBoost) algorithm. Fivefold cross-validation with early stopping was applied to optimize the number of boosting iterations and reduce overfitting. To improve reproducibility, the hyperparameter search strategy and tuning ranges used during model development are summarized in Supplementary Table S9.

For both models, the classifier generated a probability of belonging to the hyperinflammatory phenotype. Patients were classified as hyperinflammatory or hypoinflammatory using a predefined threshold of 0.5, consistent with the original study (11).

The trained 17-variable model was subsequently applied to the eICU cohort for external validation.

### Outcome definition

The primary outcome was 30-day all-cause mortality. Patients were followed from ARDS onset, defined as the baseline time point, until death or 30 days. Patients alive at 30 days were classified as survivors. Mortality status was determined using survival data recorded in the respective databases. The outcome definitions were consistently applied across both cohorts.

### Statistical analysis

Continuous variables were expressed as the median (interquartile range) and compared using the Wilcoxon rank-sum test. Categorical variables were expressed as counts (percentages) and compared using the chi-square test.

The association between inflammatory phenotypes and 30-day mortality was evaluated using logistic regression. Multivariable models were adjusted for age, sex, and a severity-of-illness score. APS III was used in the MIMIC-IV cohort, whereas the acute physiology score available in the eICU database was used in the external validation cohort because APS III is not directly available in eICU. The results were reported as odds ratios (ORs) with 95% confidence intervals (CIs).

Model discrimination was assessed using the area under the receiver operating characteristic curve. Comparisons of AUCs between the 17-variable and 18-variable models were performed using DeLong’s test in both the MIMIC-IV and eICU cohorts. The agreement between the two models was evaluated using percentage agreement.

To further assess the model’s robustness and address potential optimism bias associated with in-sample evaluation, we conducted a five-fold cross-validation analysis within the MIMIC-IV cohort. Model performance was evaluated using out-of-fold predictions generated from validation folds not involved in model fitting. The discriminatory power of the cross-validation was assessed by calculating the area under the receiver operating characteristic curve (AUC) and its corresponding 95% confidence interval (CI). Additionally, agreement, sensitivity, specificity, and Cohen’s kappa coefficient were calculated under cross-validation to evaluate the consistency between the simplified classifier and the original classifier.

To assess the robustness of the findings to missing data, multiple imputation using chained equations was performed as a sensitivity analysis in the MIMIC-IV cohort. Meanwhile, a complete-case sensitivity analysis was performed in the eICU cohort.

All analyses were performed using the R software (version 4.3.2) in a Posit environment. A two-sided *p*-value of <0.05 was considered significant.

### Ethics statement

The MIMIC-IV and eICU databases are publicly available and deidentifiable. The requirements for institutional review board approval and informed consent were waived.

An overview of the study design and analytical workflow is shown in Supplementary Figure S1.

The original 18-variable inflammatory phenotype model was validated in the MIMIC-IV cohort. Logistic regression analyses were also performed to evaluate baseline characteristics and outcomes. A simplified 17-variable model, excluding minute ventilation, was developed and compared with the original model. External validation was subsequently performed in the eICU cohort. Sensitivity analyses included multiple imputation in MIMIC-IV and complete-case analysis in the eICU cohort.

## Results

### Cohort selection and baseline characteristics

A total of 938 patients with ARDS from the MIMIC-IV cohort were included in the final analysis. Of these, 611 (65.1%) were classified as hypoinflammatory and 327 (34.9%) as hyperinflammatory. Baseline characteristics stratified by inflammatory phenotype are summarized in Table 1. No significant differences were observed in age or sex distribution between the two groups. However, patients with the hyperinflammatory phenotype exhibited more severe clinical abnormalities. Specifically, higher bilirubin and creatinine levels, lower albumin levels, and more pronounced metabolic acidosis, as reflected by lower bicarbonate levels, were observed (all *p* < 0.001). Hemodynamic instability was more pronounced in the hyperinflammatory group, as indicated by lower systolic blood pressure and a markedly higher proportion of vasopressor use (90.8% vs. 58.6%, *p* < 0.001). In addition, worse oxygenation, higher respiratory and heart rates, and reduced urine output were observed (all *p* < 0.001). Consistently, severity scores, including Sequential Organ Failure Assessment, Simplified Acute Physiology Score II, and APS III, were significantly higher in the hyperinflammatory group, indicating more severe organ dysfunction.

**Table 1:** Baseline characteristics of patients stratified by inflammatory phenotypes in the MIMIC-IV cohort.

Variable	Hypoinflammatory *N* = 611	Hyperinflammatory *N* = 327	*p*-value
Demographics			
Age, years	60.00 (48.00–70.00)	58.00 (47.00–70.00)	0.309
Male sex, *n* (%)	363 (59.4%)	185 (56.6%)	0.441
AI classifier variables			
Bilirubin, umol/L	11.97 (6.84–20.52)	32.49 (11.12–100.03)	<0.001
Creatinine, umol/L	79.56 (61.88–106.08)	176.80 (97.24–291.72)	<0.001
Albumin, g/L	29.00 (25.00–33.00)	26.00 (21.00–31.00)	<0.001
Glucose, g/L	1.34 (1.11, 1.67)	1.29 (0.97–1.71)	0.155
Sodium, mmol/L	140.00 (136.00–143.00)	138.00 (134.00–142.00)	<0.001
Hematocrit, %	31.30 (27.05–36.70)	29.90 (25.30–35.40)	0.003
White blood cell count, G/L	12.10 (8.30–17.25)	13.50 (7.70–20.45)	0.129
Platelet, G/L	190.00 (134.50–258.00)	137.00 (66.00–238.50)	<0.001
PaCO2, mmHg	44.00 (38.00–52.00)	40.00 (34.00–48.00)	<0.001
PaO2/FiO2, mmHg	187.50 (127.50–241.83)	140.00 (91.00–203.14)	<0.001
Temperature, °C	37.06 (36.56–37.61)	36.78 (36.33–37.33)	<0.001
Respiratory rate, bpm	20.00 (16.00–24.00)	24.00 (19.00–28.00)	<0.001
Heart rate, bpm	87.00 (74.00–101.00)	99.00 (81.50–115.00)	<0.001
Systolic blood pressure, mmHg	113.00 (100.00–130.00)	103.00 (92.00–116.00)	<0.001
Urine output, mL/day	1,625.00 (989.00–2,417.50)	669.00 (189.00–1,595.00)	<0.001
Minute ventilation, L/min	9.30 (7.80–11.20)	10.90 (8.70–13.00)	<0.001
Bicarbonate, mmol/L	24.00 (21.00–26.00)	18.00 (15.00–20.00)	<0.001
Vasopressor use, *n* (%)	358 (58.6%)	297 (90.8%)	<0.001
Severity scores			
SOFA score	6.00 (4.00–9.00)	12.00 (9.00–14.00)	<0.001
SAPS II score	40.00 (31.00–50.00)	55.00 (43.00–64.00)	<0.001
APS III score	53.00 (40.00–67.00)	81.00 (65.00–98.50)	<0.001
Charlson Comorbidity Index	4.00 (1.00–6.00)	4.00 (2.00–6.00)	<0.001

Continuous variables were expressed as medians (Q1, Q3), and categorical variables were expressed as *n* (%). *p*-values were calculated using the Wilcoxon rank-sum test for continuous variables and the chi-square test for categorical variables.

### Model simplification and phenotypic agreement

A simplified 17-variable model excluding minute ventilation was developed using the MIMIC-IV cohort. The distribution of inflammatory phenotypes was highly similar between the original 18-variable model and the simplified 17-variable model, accounting for 65.1%/34.9 and 65.2%/34.8% of patients, respectively. The overall agreement between the two models was 99.9%, with only one patient showing discordant classification.

**Figure 2:**
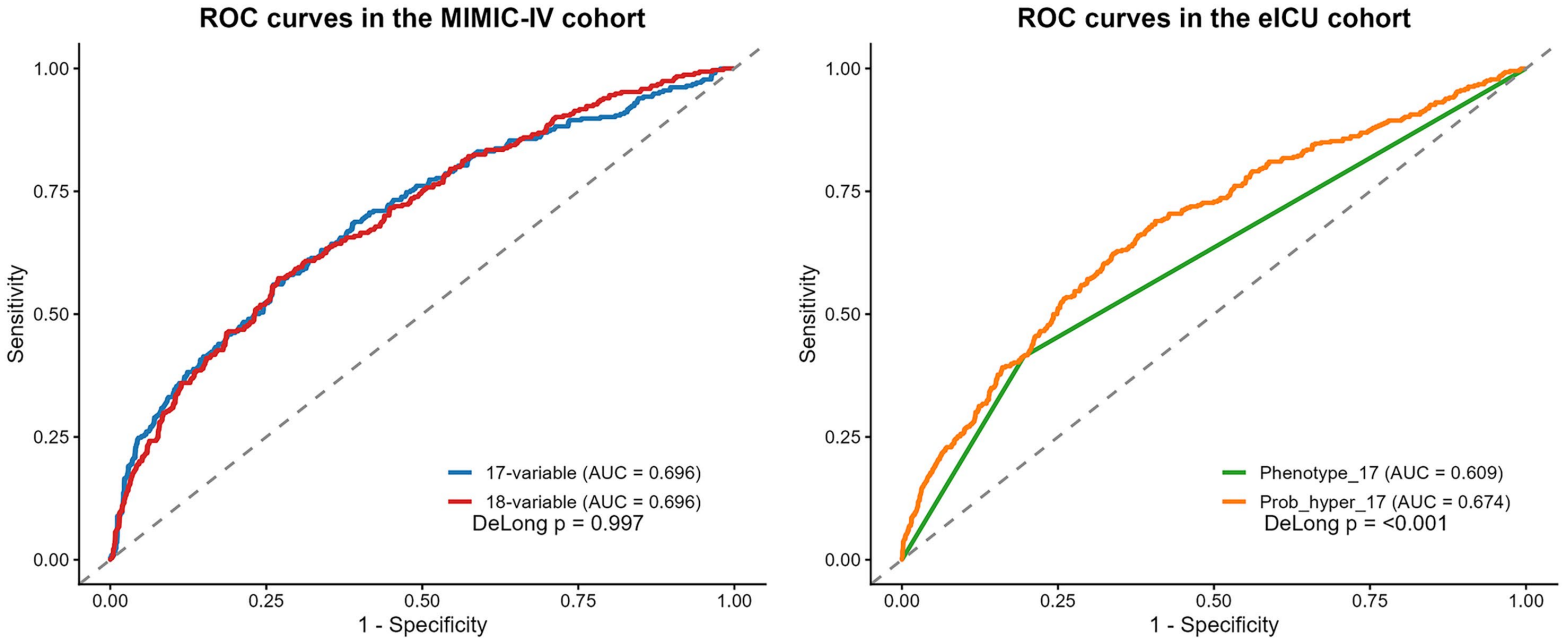
Discriminative performance of ARDS phenotype models for 30-day mortality in the MIMIC-IV and eICU cohorts. The ROC curves show the discriminative performance of the 17-variable and 18-variable models in the MIMIC-IV cohort, and the comparison between binary phenotype classification and probability-based prediction in the eICU cohort. In the MIMIC-IV cohort, the displayed AUC values were calculated using the continuous predicted probability of the hyperinflammatory phenotype generated by the classifier. Corresponding AUCs derived from binary phenotype assignments are reported separately in Table 2. Continuous model output (Prob_hyper_17) improved prognostic discrimination compared with binary classification.

The discriminative performance for 30-day mortality was also comparable between the two models, with AUCs of 0.638 and 0.636 for the 18-variable and 17-variable models, respectively. No significant difference was observed in DeLong’s test (*p* = 0.317) (Table 2). When continuous phenotype probabilities rather than binary phenotype assignments were used, both models achieved improved discrimination with identical AUCs of 0.696. In the MIMIC-IV cohort, the discriminative performance of the simplified 17-variable model remained nearly identical to that of the original 18-variable model (DeLong’s test, *p* = 0.997), as shown in Figure 2.

**Table 2:** Comparison of the 17-variable and 18-variable phenotype models in the MIMIC-IV cohort.

Variable	18-variable model	17-variable model
Phenotype distribution, *n* (%)	Hypo: 611 (65.1%)/Hyper: 327 (34.9%)	Hypo: 612 (65.2%)/Hyper: 326 (34.8%)
Overall agreement	—	99.9%
AUC for 30-day mortality	0.638	0.636
DeLong *p*-value	—	0.317
Fivefold CV: phenotype reproduction AUC (95% CI)	—	0.980 (0.973–0.987)
Fivefold CV: 30-day mortality AUC (95% CI)	—	0.686 (0.649–0.724)

AUC, area under the receiver operating characteristic curve; CI, confidence interval. Overall agreement represents the proportion of identical phenotype assignments between the simplified 17-variable model and the original 18-variable model. Fivefold cross-validation analyses were performed using out-of-fold predictions generated from validation folds not used during model fitting.

### Internal cross-validation analysis

To further evaluate the robustness of the simplified classifier and address potential optimism bias associated with in-sample evaluation, we performed five-fold cross-validation using out-of-fold predictions within the MIMIC-IV cohort. The simplified 17-variable model demonstrated excellent discriminatory ability in reproducing the phenotypic classifications generated by the original 18-variable classifier, with a cross-validation AUC of 0.980 (95% CI, 0.973–0.987). Furthermore, the model’s cross-validation AUC for 30-day mortality was 0.686 (95% CI, 0.649–0.724), comparable to the discriminatory performance observed in the primary analysis. These findings support the good stability and robustness of the simplified classifier beyond its consistency within the overall sample.

For detailed cross-validation performance metrics, including agreement, sensitivity, specificity, predictive values, and Cohen’s kappa coefficient, see Supplementary Table S4.

### Association between the inflammatory phenotypes and mortality

In the MIMIC-IV cohort, the hyperinflammatory phenotype defined by the original 18-variable model was significantly associated with increased 30-day mortality. The observed 30-day mortality rates were as follows: 24.1% for patients with the low-inflammation phenotype (147 deaths out of 611 patients) and 51.1% for patients with the high-inflammation phenotype (167 deaths out of 327 patients).

In the unadjusted analysis, the OR for mortality was 3.29 (95% CI, 2.48–4.39, *p* < 0.001). This association remained significant after adjustment for age and sex (OR: 3.73, 95% CI 2.77–5.05, *p* < 0.001) and persisted after additional adjustment for the APS III score (OR: 2.37, 95% CI 1.70–3.31, *p* < 0.001).

The results based on the simplified 17-variable model were highly consistent. The hyperinflammatory phenotype remained associated with increased mortality in the unadjusted model (OR: 3.25, 95% CI 2.45–4.33, *p* < 0.001), after adjustment for age and sex (OR: 3.67, 95% CI 2.73–4.96, *p* < 0.001), and after further adjustment for the APS III score (OR: 2.32, 95% CI 1.66–3.24, *p* < 0.001) (Table 3).

**Table 3:** Association between inflammatory phenotypes and 30-day mortality in the MIMIC-IV cohort.

Phenotype	Model	OR (95% CI)	*p*-value
Primary analysis: 18-variable phenotype			
Hyperinflammatory vs. hypoinflammatory	Unadjusted	3.29 (2.48–4.39)	<0.001
Hyperinflammatory vs. hypoinflammatory	Adjusted for age and sex	3.73 (2.77–5.05)	<0.001
Hyperinflammatory vs. hypoinflammatory	Adjusted for age, sex, and APS III	2.37 (1.70–3.31)	<0.001
Sensitivity analysis: 17-variable phenotype			
Hyperinflammatory vs. hypoinflammatory	Unadjusted	3.25 (2.45–4.33)	<0.001
Hyperinflammatory vs. hypoinflammatory	Adjusted for age and sex	3.67 (2.73–4.96)	<0.001
Hyperinflammatory vs. hypoinflammatory	Adjusted for age, sex, and APS III	2.32 (1.66–3.24)	<0.001

Odds ratios were estimated using logistic regression analysis. The primary analysis was based on the original 18-variable phenotypic model, whereas the sensitivity analysis was based on the more parsimonious 17-variable model.

### External validation in the eICU cohort

In the eICU cohort, the model demonstrated moderate discrimination for 30-day mortality. The AUC was 0.609 using the binary phenotype classification and increased to 0.674 when model-derived probabilities were used, indicating improved prognostic discrimination with continuous output (Figure 2). In the eICU cohort (1,527 patients in total, 387 of whom had a high-inflammation phenotype) and the MIMIC-IV cohort (938 patients in total, 327 of whom had a high-inflammation phenotype), the prevalence of the high-inflammation phenotype was lower in the eICU cohort than in the MIMIC-IV cohort (25.3% vs. 34.8%). In addition, differences were observed in several baseline characteristics, including disease severity, use of vasopressors, and mortality. A detailed comparison of baseline characteristics between the two cohorts is provided in Supplementary Table S5.

### Performance and discrimination of the model

The external validation cohort included 1,527 patients with ARDS from the eICU database. Using the 17-variable model, 1,140 patients (74.7%) were classified as hypoinflammatory and 387 (25.3%) as hyperinflammatory.

The hyperinflammatory phenotype was associated with a significantly higher 30-day mortality rate (43.4% vs. 20.9%, respectively). In the unadjusted analysis, the OR for mortality was 2.91 (95% CI 2.27–3.72, *p* < 0.001). The association remained significant after adjustment for age, sex, and acute physiology score (OR: 1.62, 95% CI 1.21–2.15, *p* < 0.001).

The AUC for 30-day mortality was 0.674 when the predicted probability was used as a predictor (Table 4).

**Table 4:** Summary of external validation of the 17-variable model in the eICU cohort.

Variable	Value
Phenotype distribution, *n* (%)	Hypo: 1140 (74.7%)/Hyper: 387 (25.3%)
30-day mortality by phenotype, %	20.9% vs. 43.4%
Unadjusted OR (95% CI)	2.91 (2.27–3.72)
Adjusted OR (95% CI)	1.62 (1.21–2.15)
AUC for 30-day mortality	0.674

The adjusted odds ratios were derived from a multivariable model including age, sex, and acute physiology score. The AUC was calculated using prob_hyper_17 as the predictor for 30-day mortality.

### Sensitivity analyses

Sensitivity analyses confirmed the robustness of the findings. In the MIMIC-IV cohort, multiple imputations yielded consistent results. Five multiply imputed datasets were generated using chained equations. The proportion of the hyperinflammatory phenotype ranged from 34.8 to 35.7% across the five imputed datasets, indicating highly stable phenotype assignment (Supplementary Table S1). The association between the hyperinflammatory phenotype and mortality remained significant in the pooled analysis (OR: 2.59, 95% CI: 1.83–3.66), and the effect estimate was comparable to that observed in the primary analysis, further supporting the robustness of the findings (Supplementary Table S2).

In the eICU cohort, complete-case analysis yielded consistent results. Among 954 patients with complete data, the hyperinflammatory phenotype remained associated with increased mortality (adjusted OR: 1.62, 95% CI: 1.18–2.23). The model discrimination was comparable to the primary analysis (Supplementary Table S3).

Detailed summaries of variable-specific missingness are provided in Supplementary Table S10 for the MIMIC-IV cohort and Supplementary Table S6 for the eICU cohort. A comparison between patients included and excluded from the eICU full-case analysis is provided in Supplementary Table S7.

## Discussion

In this study, a machine learning–based inflammatory phenotype classifier was validated in a large ICU cohort. A simplified version excluding minute ventilation was evaluated. Three main findings were identified. First, the hyperinflammatory phenotype was consistently associated with increased 30-day mortality, independent of illness severity. Second, the simplified 17-variable model demonstrated near-perfect agreement with the original model while preserving the prognostic performance. Third, these findings were reproducible in an external multicenter cohort, supporting the generalizability of the model.

The observed association between the hyperinflammatory phenotype and increased mortality is consistent with the findings of previous studies on ARDS subphenotypes (13, 14). Notably, this association remained significant after adjusting for severity scores. This finding suggests that inflammatory phenotypes capture prognostic information beyond conventional clinical indices (15).

Although the present study focused on phenotype classification at ARDS onset, emerging evidence suggests that inflammatory phenotypes may evolve dynamically over time, which could further influence prognosis and treatment responsiveness (8, 16, 17). Future studies incorporating longitudinal phenotype assessment may provide additional insights into phenotype-guided management strategies.

A key contribution of this study is the identification of minute ventilation as a barrier to real-world implementation and the demonstration that removing this variable does not compromise the classifier’s performance. Although minute ventilation was included in the original model, ventilatory variables may be more difficult to capture consistently from routine electronic health record systems than laboratory and vital sign variables and may also be influenced by local ventilation management practices. Previous studies have highlighted that ventilatory variables can present challenges for reliable EHR-based implementation of ARDS phenotype classifiers, potentially limiting model portability across institutions (12). Unlike laboratory and physiological variables, which primarily reflect patient characteristics, minute ventilation may partially reflect clinical management decisions. Therefore, reliance on this variable may reduce the model’s transferability across different institutions. The high consistency observed between the 17-variable and 18-variable models suggests that removing this implementation-sensitive variable can enhance the model’s practicality and transferability without causing a meaningful loss of prognostic information. This finding is consistent with previous studies showing that parsimonious models based on readily available clinical data can achieve performance similar to more complex approaches (9, 10).

Our study also differs from previously published simplified phenotypic classifiers. Sinha and colleagues developed simplified algorithms to classify ARDS phenotypes derived from latent class analysis and validated these models primarily in clinical trial populations (9). Similar to these parsimonious approaches, our simplified classifier was designed to improve clinical applicability by reducing reliance on variables that may not be routinely available in all healthcare systems. Importantly, the simplified 17-variable model maintained near-perfect agreement with the original classifier and preserved prognostic performance, supporting the feasibility of model simplification without substantial loss of information. In contrast, this study focuses on the external validation of a previously published machine-learning-based clinical classifier in a large real-world ICU database (11) and evaluates whether simplifications aimed at practical application can retain the classifier’s utility for phenotypic classification and prognostic prediction across diverse, heterogeneous healthcare systems. Therefore, the contribution of our work lies not in developing yet another simplified classifier, but in demonstrating that existing AI-based classifiers can be adapted for broader clinical deployment while maintaining their performance.

External validation in an eICU cohort further strengthened the robustness of our findings (7). Validation across independent datasets is essential for assessing model transportability and real-world applicability, particularly in heterogeneous critical care populations. Despite differences in patient populations and data structures, the association between the hyperinflammatory phenotype and mortality remained consistent.

It is worth noting that there was a difference in the prevalence of the high-inflammation phenotype between the MIMIC-IV cohort and the eICU cohort (34.8% vs. 25.3%). Several factors may explain this finding. First, differences in ARDS diagnosis are inevitable because the eICU database lacks continuous access to the chest imaging data required for strict Berlin definition-based diagnosis. Consequently, a pragmatic ARDS identification strategy was adopted in the external validation cohort, which may have led to differences in case composition. Second, significant differences exist between MIMIC-IV and eICU in terms of data structure, participating institutions, clinical practice patterns, and patient populations. Third, baseline differences were observed between the two cohorts regarding disease severity, vasopressor use, and mortality (Supplementary Table S5), which may also have contributed to variations in the prevalence of the inflammatory phenotype. Despite these differences, the association between the high-inflammation phenotype and adverse outcomes remained consistent across cohorts, supporting the classifier’s generalizability and robustness.

Although the extent of potential diagnostic misclassification could not be directly quantified because imaging data were unavailable in eICU, the preservation of mortality associations and model discrimination across cohorts suggests that any resulting bias was unlikely to substantially alter the main conclusions.

The mortality discrimination observed in the present study is broadly consistent with previous ARDS phenotyping investigations. Similar studies based on latent class analysis and machine-learning approaches have generally reported modest to moderate prognostic discrimination while emphasizing biological heterogeneity, treatment responsiveness, and subgroup identification as their primary objectives. Therefore, the AUC values observed in this study should not be interpreted as the performance of a dedicated mortality prediction model but rather as evidence that the identified inflammatory phenotypes retain clinically meaningful prognostic information.

From a clinical perspective, the simplified model relies exclusively on routinely available variables. It does not require specialized biomarkers, thereby facilitating implementation across diverse healthcare settings. The rapid identification of inflammatory phenotypes may support bedside risk stratification and inform precision-based medical strategies for ARDS. Accumulating evidence suggests that treatment responses, particularly to corticosteroids, may differ across phenotypes. This observation highlights the potential for phenotype-guided therapeutic strategies (18).

This study has some limitations. First, a retrospective study was used, and residual confounding factors could not be excluded. Second, both datasets were derived from ICU populations in the United States and therefore primarily reflect clinical practice patterns, healthcare resources, and patient characteristics in high-income Western healthcare systems. Consequently, the generalizability of the findings to non-Western regions and low- and middle-income countries may be limited. Further validation in geographically diverse and resource-limited ICU populations is warranted. In addition, complete-case analysis was used in the eICU cohort because of differences in missingness patterns across participating institutions. Comparison of included and excluded patients demonstrated differences in illness severity and mortality (Supplementary Table S7), suggesting that missingness was not completely random and that some degree of selection bias cannot be excluded. Nevertheless, the complete-case analysis yielded results consistent with the primary analysis, supporting the robustness of the overall findings. In addition, vasopressor use was included as a baseline clinical support variable in the classifier. Because vasopressor administration may be influenced by evolving inflammatory status and clinician treatment decisions, some degree of reverse causation or treatment-related bias cannot be completely excluded. Third, the model demonstrated only moderate discrimination for mortality, reflecting its primary role as a phenotyping tool rather than as a dedicated outcome prediction model. The classifier was developed to identify clinically meaningful inflammatory phenotypes rather than to maximize prediction of mortality risk. Therefore, 30-day mortality discrimination was evaluated as a clinically relevant secondary measure to assess whether the simplified classifier preserved the prognostic utility of the original phenotype assignments. Accordingly, model performance should be interpreted within a phenotyping framework rather than according to the standards typically applied to dedicated prognostic models (19). Finally, although differential treatment response across inflammatory phenotypes may represent a more direct measure of phenotyping utility, evaluation of treatment-effect heterogeneity was beyond the scope of the present validation study and warrants further investigation in future prospective studies.

## Conclusion

This inflammatory phenotype classifier provides robust and generalizable prognostic information for critically ill patients with ARDS. A simplified model excluding minute ventilation retains comparable performance. It also improves feasibility, supporting its potential for real-world clinical implementation.

## Data Availability

Publicly available datasets were analyzed in this study. This data can be found at: Publicly available datasets were analyzed in this study. The datasets can be found through the PhysioNet repository. MIMIC-IV database: https://physionet.org/content/mimiciv/ eICU Collaborative Research Database: https://physionet.org/content/eicu-crd/ access to both databases requires completion of the required credentialing process and data use agreements via PhysioNet.

## References

[1] Al-Husinat L Azzam S Al Sharie S Araydah M Battaglini D Abushehab S . A narrative review on the future of ARDS: evolving definitions, pathophysiology, and tailored management. Crit Care. (2025) 29:88. doi: 10.1186/s13054-025-05291-0, 39994815 PMC11852867

[2] ARDS Definition Task Force Ranieri VM Rubenfeld GD Thompson BT Ferguson ND Caldwell E . Acute respiratory distress syndrome: the Berlin definition. JAMA. (2012) 307:2526–33. doi: 10.1001/jama.2012.5669, 22797452

[3] Matthay M Arabi Y Arroliga A Bernard G Bersten A Brochard L . A new global definition of acute respiratory distress syndrome. Am J Respir Crit Care Med. (2023) 209:37–47. doi: 10.1164/rccm.202303-0558ws, 37487152 PMC10870872

[4] Gonzalez-Pizarro P Suárez-Sipmann F. Acute respiratory distress syndrome definitions in adults and children: a comparative narrative review. J Clin Med. (2025) 14:7644. doi: 10.3390/jcm14217644, 41227040 PMC12607997

[5] Sinha P Spicer A Delucchi KL McAuley DF Calfee CS Churpek MM. Comparison of machine learning clustering algorithms for detecting heterogeneity of treatment effect in acute respiratory distress syndrome: a secondary analysis of three randomised controlled trials. EBioMedicine. (2021) 74:103697. doi: 10.1016/j.ebiom.2021.103697, 34861492 PMC8645454

[6] Sathe NA Morrell ED Bhatraju PK Fessler MB Stapleton RD Wurfel MM . Alveolar biomarker profiles in subphenotypes of the acute respiratory distress syndrome. Crit Care Med. (2023) 51:e13–8. doi: 10.1097/CCM.0000000000005704, 36519995 PMC9764239

[7] Liu X Jiang Y Jia X Ma X Han C Guo N . Identification of distinct clinical phenotypes of acute respiratory distress syndrome with differential responses to treatment. Crit Care. (2021) 25:320. doi: 10.1186/s13054-021-03734-y, 34461969 PMC8404019

[8] Delucchi K Famous KR Ware LB Parsons PE Thompson BT Calfee CS. Stability of ARDS subphenotypes over time in two randomised controlled trials. Thorax. (2018) 73:439–45. doi: 10.1136/thoraxjnl-2017-211090, 29477989 PMC6497167

[9] Sinha P Delucchi KL McAuley DF O’Kane CM Matthay MA Calfee CS. Development and validation of parsimonious algorithms to classify acute respiratory distress syndrome phenotypes: a secondary analysis of randomised controlled trials. Lancet Respir Med. (2020) 8:247–57. doi: 10.1016/S2213-2600(19)30369-8, 31948926 PMC7543720

[10] Sinha P Churpek MM Calfee CS. Machine learning classifier models can identify acute respiratory distress syndrome phenotypes using readily available clinical data. Am J Respir Crit Care Med. (2020) 202:996–1004. doi: 10.1164/rccm.202002-0347OC, 32551817 PMC7528785

[11] Pensier J Fosset M Paschold B-S Von Wedel D Redaelli S Braeuer BLP . Temporal stability of phenotypes of acute respiratory distress syndrome: clinical implications for early corticosteroid therapy and mortality. Intensive Care Med. (2025) 51:1784–96. doi: 10.1007/s00134-025-08089-4 40839098

[12] Maddali MV Churpek M Pham T Rezoagli E Zhuo H Zhao W. Validation and utility of ARDS subphenotypes identified by machine learning models using clinical data: an observational multi-cohort retrospective analysis. Lancet Respir Med. (2022) 10:367–77. doi: 10.1016/S2213-2600(21)00461-6, 35026177 PMC8976729

[13] Famous KR Delucchi K Ware LB Kangelaris KN Liu KD Thompson BT . Acute respiratory distress syndrome subphenotypes respond differently to randomized fluid management strategy. Am J Respir Crit Care Med. (2017) 195:331–8. doi: 10.1164/rccm.201603-0645OC, 27513822 PMC5328179

[14] Sinha P Delucchi KL Thompson BT McAuley DF Matthay MA. Latent class analysis of ARDS subphenotypes: a secondary analysis of the statins for acutely injured lungs from Sepsis (SAILS) study. Intensive Care Med. (2018) 44:1859–69. doi: 10.1007/s00134-018-5378-3, 30291376 PMC6317524

[15] Calfee CS Delucchi K Parsons PE Thompson BT Ware LB Matthay MA. Subphenotypes in acute respiratory distress syndrome: latent class analysis of data from two randomised controlled trials. Lancet Respir Med. (2014) 2:611–20. doi: 10.1016/S2213-2600(14)70097-9, 24853585 PMC4154544

[16] Slim MA van Amstel RBE Bos LDJ Cremer OL Wiersinga WJ van der Poll T . Inflammatory subphenotypes previously identified in ARDS are associated with mortality at intensive care unit discharge: a secondary analysis of a prospective observational study. Crit Care. (2024) 28:151. doi: 10.1186/s13054-024-04929-9, 38715131 PMC11077885

[17] Chen H Yu Q Xie J Liu S Pan C Liu L . Longitudinal phenotypes in patients with acute respiratory distress syndrome: a multi-database study. Crit Care. (2022) 26:340. doi: 10.1186/s13054-022-04211-w, 36333766 PMC9635207

[18] Grasselli G Calfee CS Camporota L Poole D Amato MBP Antonelli M . ESICM guidelines on acute respiratory distress syndrome: definition, phenotyping and respiratory support strategies. Intensive Care Med. (2023) 49:727–59. doi: 10.1007/s00134-023-07050-7, 37326646 PMC10354163

[19] Collins GS Reitsma JB Altman DG Moons KGM. Transparent reporting of a multivariable prediction model for individual prognosis or diagnosis (TRIPOD): the TRIPOD statement. BMJ. (2015) 350:g7594–4. doi: 10.1136/bmj.g7594, 25569120

