# Supplementary material for: Inflammatory phenotypes in acute respiratory distress syndrome: external validation and model simplification in multicenter cohorts
Source: Front Med (Lausanne). 2026 Jun 24;13:1877882. doi: 10.3389/fmed.2026.1877882 (PMC13342228; doi:10.3389/fmed.2026.1877882)
Supplement: Supplementary file 1 [file Supplementary_file_1.docx]

Supplementary Material

# Supplementary Data

Supplementary Material should be uploaded separately on submission. Please include any supplementary data, figures and/or tables.

Supplementary material is not typeset so please ensure that all information is clearly presented, the appropriate caption is included in the file and not in the manuscript, and that the style conforms to the rest of the article.

# Supplementary Figures and Tables

## Supplementary Figures

**
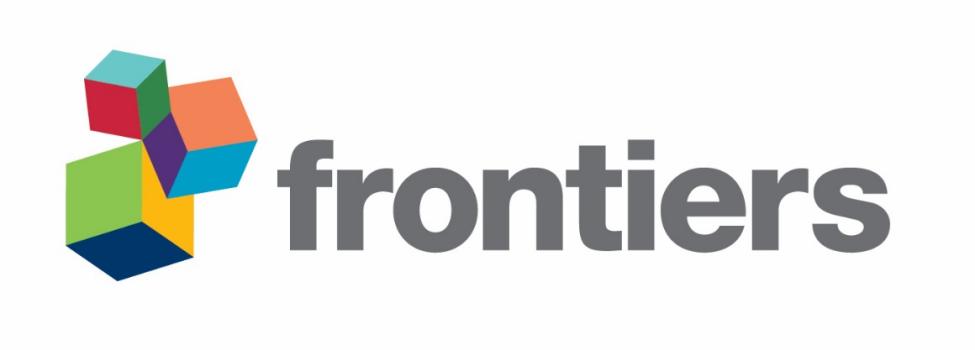
**

**Supplementary Fig. 1**

Study design and analytical framework.


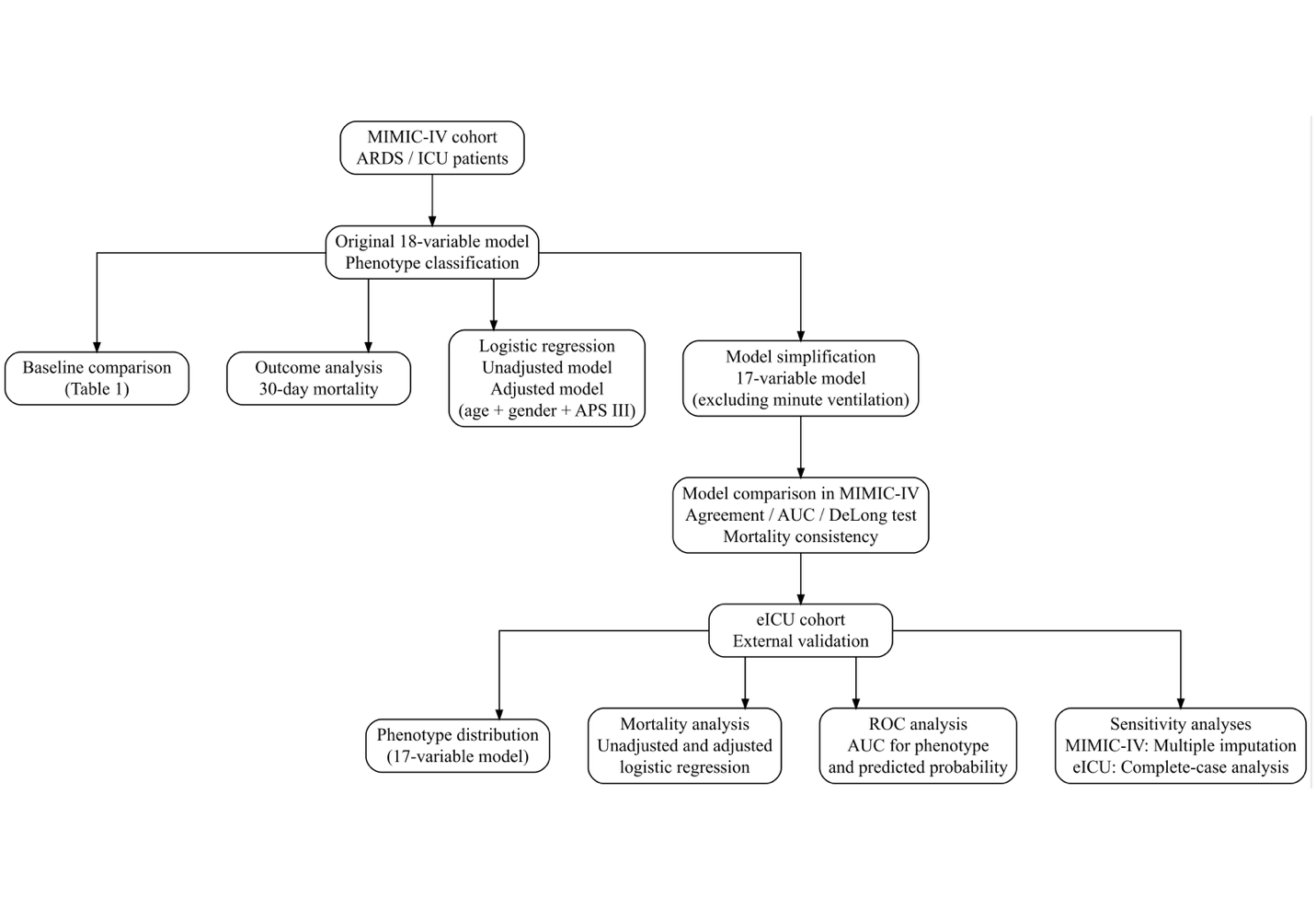


**Supplementary Table S1**

Distribution of inflammatory phenotypes across the five imputed datasets

| **Imputed dataset** | **Hypoinflammatory, n (%)** | **Hyperinflammatory, n (%)** |
| --- | --- | --- |
| 1 | 609 (64.9%) | 329 (35.1%) |
| 2 | 607 (64.7%) | 331 (35.3%) |
| 3 | 610 (65.0%) | 328 (35.0%) |
| 4 | 603 (64.3%) | 335 (35.7%) |
| 5 | 612 (65.2%) | 326 (34.8%) |

Values are presented as n (%).

**Supplementary Table S2**.

Association between the 18-variable inflammatory phenotype and 30-day mortality after multiple imputation

| **Sensitivity analysis** | **Adjusted OR (95% CI)** |
| --- | --- |
| Multiple imputation sensitivity analysis (18-variable phenotype) | 2.59 (1.83–3.66) |

Odds ratios were pooled across the imputed datasets using Rubin’s rules. The fully adjusted model included age, sex, and APS III score.

**Supplementary Table S3**.

Summary of complete-case sensitivity analysis in the eICU cohort

| **Variable** | **Value** |
| --- | --- |
| Complete-case sample size | 954 |
| Phenotype distribution, n (%) | Hypo: 637 (66.8%) / Hyper: 317 (33.2%) |
| 30-day mortality by phenotype, % | 25.1% vs 46.4% |
| Unadjusted OR (95% CI) | 2.58 (1.94–3.43) |
| Adjusted OR (95% CI) | 1.62 (1.18–2.23) |
| AUC using phenotype_17 | 0.608 |
| AUC using prob_hyper_17 | 0.670 |

Complete-case analysis included only patients with complete data for all 17 model variables, age, sex, acute physiology score, and 30-day mortality.

**Supplementary Table S4**

Supplementary Table S4. Fivefold cross-validation performance of the simplified 17-variable classifier in the MIMIC-IV cohort

| **Metric** | **Value** |
| --- | --- |
| Sample size | 938 |
| Cross-validated agreement with 18-variable model | 92.2% |
| Accuracy | 92.2% |
| Cohen’s kappa | 0.827 |
| Sensitivity for hyperinflammatory phenotype | 86.2% |
| Specificity for hyperinflammatory phenotype | 95.4% |
| Positive predictive value | 91.0% |
| Negative predictive value | 92.8% |
| Cross-validated AUC for reproducing 18-variable phenotype | 0.980 (95% CI, 0.973–0.987) |
| Cross-validated AUC for 30-day mortality | 0.686 (95% CI, 0.649–0.724) |
| Unadjusted OR for 30-day mortality using CV phenotype | 3.50 (95% CI, 2.63–4.68), p < 0.001 |

AUC, area under the receiver operating characteristic curve; CI, confidence interval; CV, cross-validation; OR, odds ratio. Cross-validation performance was evaluated using out-of-fold predictions generated from fivefold cross-validation within the MIMIC-IV cohort. Agreement, accuracy, sensitivity, specificity, predictive values, and Cohen’s kappa were calculated relative to phenotype assignments generated by the original 18-variable classifier.

# Supplementary Table S5

Comparison of baseline characteristics between the MIMIC-IV and eICU cohorts

| **Variable** | **Overall**^1^ | **MIMIC-IV**^1^ | **eICU**^1^ | **p-value**^2^ |
| --- | --- | --- | --- | --- |
| **Age, years** |  |  |  | 0.3 |
| Median (Q1–Q3) | 60.00 (48.00–71.00) | 59.00 (47.00–70.00) | 61.00 (50.00–71.00) |  |
| **Male sex** |  |  |  | 0.086 |
| Female | 970.0 (39.4%) | 390.0 (41.6%) | 580.0 (38.0%) |  |
| Male | 1,494.0 (60.6%) | 548.0 (58.4%) | 946.0 (62.0%) |  |
| **Inflammatory phenotype** |  |  |  | <0.001 |
| Hypoinflammatory | 1,752.0 (71.1%) | 612.0 (65.2%) | 1,140.0 (74.7%) |  |
| Hyperinflammatory | 713.0 (28.9%) | 326.0 (34.8%) | 387.0 (25.3%) |  |
| **30-day mortality** |  |  |  | <0.001 |
| Survivor | 1,745.0 (70.8%) | 624.0 (66.5%) | 1,121.0 (73.4%) |  |
| Non-survivor | 720.0 (29.2%) | 314.0 (33.5%) | 406.0 (26.6%) |  |
| **Severity score** |  |  |  | <0.001 |
| Median (Q1–Q3) | 67.00 (48.00–90.00) | 61.00 (45.00–81.00) | 72.00 (51.00–97.00) |  |
| **PaO2/FiO2 ratio, mmHg** |  |  |  | 0.9 |
| Median (Q1–Q3) | 170.00 (112.50–235.00) | 168.17 (114.00–234.00) | 170.77 (111.25–235.00) |  |
| **Vasopressor use** |  |  |  | <0.001 |
| No | 961.0 (39.0%) | 283.0 (30.2%) | 678.0 (44.4%) |  |
| Yes | 1,504.0 (61.0%) | 655.0 (69.8%) | 849.0 (55.6%) |  |
| **Creatinine, umol/L** |  |  |  | 0.4 |
| Median (Q1–Q3) | 1.10 (0.79–1.82) | 1.10 (0.80–1.80) | 1.11 (0.78–1.84) |  |
| **Bilirubin, umol/L** |  |  |  | 0.008 |
| Median (Q1–Q3) | 0.80 (0.50–1.80) | 0.90 (0.40–2.20) | 0.80 (0.50–1.50) |  |
| **Platelet, G/L** |  |  |  | 0.14 |
| Median (Q1–Q3) | 171.00 (110.00–241.00) | 176.00 (110.00–251.00) | 169.00 (110.00–235.00) |  |
| **White blood cell count, G/L** |  |  |  | 0.5 |
| Median (Q1–Q3) | 12.60 (8.50–18.00) | 12.55 (8.20–18.30) | 12.60 (8.70–17.80) |  |
| ^1^n (%) | | | | |
| ^2^Wilcoxon rank sum test; Pearson's Chi-squared test | | | | |

Values are expressed as median (interquartile range) or n (%). The severity score represents APS III in the MIMIC-IV cohort and Acute Physiology Score in the eICU cohort. Because these scores are not identical measures, comparisons should be interpreted descriptively.p-values were calculated using the Wilcoxon rank-sum test for continuous variables and the chi-square test for categorical variables. ARDS, acute respiratory distress syndrome; eICU, electronic intensive care unit; MIMIC-IV, Medical Information Mart for Intensive Care IV.

# Supplementary Table S6

Missingness of key variables in the eICU cohort

| **Variable** | **Missing_n** | **Missing_percent** |
| --- | --- | --- |
| Albumin | 451 | 29.5 |
| Bilirubin | 439 | 28.7 |
| Acute physiology score | 112 | 7.3 |
| Respiratory rate | 29 | 1.9 |
| Urine output | 5 | 0.3 |
| Sodium | 1 | 0.1 |
| White blood cell count | 2 | 0.1 |
| Heart rate | 2 | 0.1 |
| Systolic blood pressure | 2 | 0.1 |
| Bicarbonate | 2 | 0.1 |
| Creatinine | 0 | 0.0 |
| Glucose | 0 | 0.0 |
| Hematocrit | 0 | 0.0 |
| Platelet | 0 | 0.0 |
| PaCO2 | 0 | 0.0 |
| PaO2/FiO2 ratio | 0 | 0.0 |
| Temperature | 0 | 0.0 |
| Vasopressor use | 0 | 0.0 |
| Age | 0 | 0.0 |
| Sex | 0 | 0.0 |
| 30-day mortality | 0 | 0.0 |

Values are presented as number and percentage of missing observations. Variables include the 17 simplified classifier variables and covariates used in the eICU complete-case sensitivity analysis.

# Supplementary Table S7

Comparison between included and excluded patients in the eICU complete-case analysis

| **Variable** | **Included**^1^ | **Excluded**^1^ | **p-value**^2^ |
| --- | --- | --- | --- |
| **Age, years** |  |  | 0.4 |
| Median (Q1–Q3) | 60.00 (50.00–70.00) | 61.00 (50.00–72.00) |  |
| **Male sex** |  |  | 0.4 |
| Female | 371.0 (38.9%) | 209.0 (36.5%) |  |
| Male | 583.0 (61.1%) | 363.0 (63.5%) |  |
| **Acute physiology score** |  |  | <0.001 |
| Median (Q1–Q3) | 78.00 (58.00–103.00) | 59.00 (41.00–80.00) |  |
| **30-day mortality** |  |  | <0.001 |
| Survivor | 648.0 (67.9%) | 473.0 (82.7%) |  |
| Non-survivor | 307.0 (32.1%) | 99.0 (17.3%) |  |
| **Vasopressor use** |  |  | <0.001 |
| No | 364.0 (38.1%) | 314.0 (54.9%) |  |
| Yes | 591.0 (61.9%) | 258.0 (45.1%) |  |
| ^1^n (%) | | | |
| ^2^Wilcoxon rank sum test; Pearson's Chi-squared test | | | |

Values are expressed as median (interquartile range) or n (%). Included patients were those with complete data for all 17 simplified classifier variables, age, sex, acute physiology score, and 30-day mortality. p-values were calculated using the Wilcoxon rank-sum test for continuous variables and the chi-square test for categorical variables.

Supplementary Table S8

Variable extraction protocol for the original 18-variable classifier and the simplified 17-variable classifier

| Variable | Source | Window | Rule | Unit |
| --- | --- | --- | --- | --- |
| Albumin | Laboratory records | ±24 h around ARDS onset | Closest available value | g/L |
| Bilirubin | Laboratory records | ±24 h around ARDS onset | Closest available value | μmol/L |
| Creatinine | Laboratory records | ±24 h around ARDS onset | Closest available value | μmol/L |
| Glucose | Laboratory records | ±24 h around ARDS onset | Closest available value | mmol/L |
| Sodium | Laboratory records | ±24 h around ARDS onset | Closest available value | mmol/L |
| Hematocrit | Laboratory records | ±24 h around ARDS onset | Closest available value | % |
| White blood cell count | Laboratory records | ±24 h around ARDS onset | Closest available value | ×10⁹/L |
| Platelet count | Laboratory records | ±24 h around ARDS onset | Closest available value | ×10⁹/L |
| PaCO2 | Blood gas | ±24 h around ARDS onset | Closest available value | mmHg |
| PaO2/FiO2 ratio | Blood gas | ±24 h around ARDS onset | Closest available value | mmHg |
| Temperature | Vital signs | ±24 h around ARDS onset | Closest available value | °C |
| Respiratory rate | Vital signs | ±24 h around ARDS onset | Closest available value | breaths/min |
| Heart rate | Vital signs | ±24 h around ARDS onset | Closest available value | bpm |
| Systolic blood pressure | Vital signs | ±24 h around ARDS onset | Closest available value | mmHg |
| Urine output | Output records | ±24 h around ARDS onset | Cumulative urine output | mL/day |
| Bicarbonate | Laboratory records | ±24 h around ARDS onset | Closest available value | mmol/L |
| Vasopressor use | Medication records | ±24 h around ARDS onset | Any administration | Binary |
| Minute ventilation* | Ventilator records | ±24 h around ARDS onset | Closest available value | L/min |

# Supplementary Table S9

Hyperparameter search strategy used for training the simplified 17-variable XGBoost classifier

| **Hyperparameter** | **Value** |
| --- | --- |
| Algorithm | Extreme gradient boosting (XGBoost) |
| Model type | xgbTree |
| Outcome | Hyperinflammatory phenotype label |
| Evaluation metric | ROC AUC |
| Validation strategy | Five-fold cross-validation |
| Early stopping | Applied |
| nrounds | 100, 200, 500 |
| max_depth | 2, 3, 4, 5 |
| eta | 0.01, 0.05, 0.10 |
| gamma | 0 |
| colsample_bytree | 1 |
| min_child_weight | 1 |
| subsample | 1 |
| Final model object | xgb.Booster |
| Final boosting iterations | 199 |

Note: Hyperparameters were evaluated using a predefined grid-search strategy. Five-fold cross-validation with early stopping was applied during model development. The final classifier consisted of 199 boosting iterations. ROC AUC, area under the receiver operating characteristic curve.

Supplementary Table S10

Missingness of variables included in the inflammatory phenotype classifier in the MIMIC-IV cohort

| **Variable** | **Missing_n** | **Missing_percent** |
| --- | --- | --- |
| Albumin | 451 | 48.1 |
| Bilirubin | 261 | 27.8 |
| Temperature | 31 | 3.3 |
| Urine output | 10 | 1.1 |
| Creatinine | 0 | 0.0 |
| Glucose | 0 | 0.0 |
| Sodium | 0 | 0.0 |
| Hematocrit | 0 | 0.0 |
| White blood cell count | 0 | 0.0 |
| Platelet count | 0 | 0.0 |
| PaCO2 | 0 | 0.0 |
| PaO2/FiO2 ratio | 0 | 0.0 |
| Respiratory rate | 0 | 0.0 |
| Heart rate | 0 | 0.0 |
| Systolic blood pressure | 0 | 0.0 |
| Bicarbonate | 0 | 0.0 |
| Vasopressor use | 0 | 0.0 |
| Minute ventilation | 0 | 0.0 |

Note: Missingness was calculated in the MIMIC-IV cohort before sensitivity analyses. Variables listed include those used in the original 18-variable classifier and the simplified 17-variable classifier.
